# Angiographic prostatic arterial anatomy in a Turkish population with benign prostatic hyperplasia

**DOI:** 10.3906/sag-2004-289

**Published:** 2021-04-30

**Authors:** Fatma Gonca ELDEM, Fırat ATAK, Osman ÖCAL, Ali Cansu BOZACI, Ahmet GÜDELOĞLU, Bora PEYNİRCİOĞLU

**Affiliations:** 1 Department of Radiology, Faculty of Medicine, Hacettepe University, Ankara Turkey; 2 Department of Urology, Faculty of Medicine, Hacettepe University, Ankara Turkey

**Keywords:** Prostate artery embolization, prostatic arterial supply, interventional radiology

## Abstract

**Background/aim:**

Prostatic artery embolization (PAE) is a minimally invasive effective method in the treatment of benign prostatic hyperplasia (BPH). The procedure is technically challenging, as pelvic vascular anatomy is highly prone to variations and the identification of the prostatic artery (PA) is the most time-consuming step, which can lead to increased procedure times. The aim of this study was to categorize the anatomic variations in the prostatic supply in patients with BPH treated with PAE.

**Materials and methods:**

The digital subtraction angiography findings of 68 PAE procedures were reviewed retrospectively and the age, PA origin, number, and procedure of the patients were recorded. The origin of the PA was classified into 5 subtypes using the de Assis/Carnavale classification. The incidence of each anatomic type was calculated.

**Results:**

In the 68 PAE procedures, 119 pelvic sides were analyzed and a total of 119 PAs were classified. The most common origin was type 1 (n = 43, 36.1%), with the PA originating from the anterior division of the internal iliac artery (IIA), from a common trunk with the superior vesical artery. This was followed by type 4 (n = 34, 28.6%), with the PA originating from the internal pudendal artery; type 3 (n = 22, 18.5%), with the PA originating from the obturator artery; and type 2 (n = 13, 10.9%), with the PA originating from the anterior division of the IIA.

**Conclusion:**

Anatomic variations are common in the IIA and PA, showing racial and individual differences. Following a standard classification system to identify the origin of the PA is crucial and being aware of the most common types in each population will make PAE a faster and safer procedure.

## 1. Introduction

Prostatic artery embolization (PAE) is a safe and effective method in the treatment of benign prostatic hyperplasia (BPH) [1,2]. The procedure is technically challenging due to anatomic aspects, which can lead to increased procedure time, thus resulting in increased radiation exposure for both the patients and operators. The most compelling part of PAE is to identify the prostatic arteries (PAs) to avoid complications of nontarget embolization [3]. 

The multiple origins of PAs were described in cadaveric studies many years ago [4–6]. However, with the invention of PAE, the renewed interest in the prostatic supply resulted in studies that have defined the radiologic anatomy of the male pelvis and PAs using digital subtraction angiography (DSA), computer tomography angiography, and magnetic resonance imaging angiography. As many anatomic variations are seen in male pelvic vascular anatomy [7], PAs are also highly prone to these variations and might show racial differences in different populations. Herein, the anatomical differences in the prostatic supply of a Turkish population were described and categorized so as to supplement and compare them with previous studies. 

## 2. Materials and methods

After approval from the institutional review board, the DSA findings from 68 PAE procedures (136 pelvic sides), performed at our institution between January 2014 and December 2018, were reviewed, retrospectively. Indications for PAE were as follows: 1) lower urinary tract symptoms due to BPH refractory or intolerant to medical treatment and 2) infravesical obstruction with acute urinary retention due to BPH. Informed consent was obtained from all of the patients. Procedures were performed according to previously described techniques [8,9], under intravenous sedation, with the intention of embolizing the uni/bilateral prostatic arteries with 100–300 µ and/or 300–500 µ trisacyrilic gelatin microspheres (Meritt Medical, South Jordan, UT, USA) to complete stasis.

Digital angiography was first performed in the aorta to visualize both pelvic sides and the internal iliac arteries (IIAs) (injection volume 15 mL, injection rate 15 mL/s). Afterwards, the contralateral IIA was selectively catheterized with a cobra-shaped catheter (C2 4F) and digital angiography (injection volume 10 mL, injection rate 5 mL/s) was performed in left anterior oblique projection (35–45°) and caudal-cranial angulation (10°) by the single femoral approach. The ipsilateral IIA was catheterized by creating a Waltman loop on the Cobra catheter or with a reversed curved catheter (Simmons 4F), and the same injection rates with right anterior oblique projection (35–45°) and caudal-cranial angulation (10°) were applied. Afterwards, the prostatic arteries were selectively catheterized with a microcatheter (2.0F Progreat, Terumo) and DSA was performed via hand injections (1–2.5 mL syringes), in both anterior oblique and neutral projections, to visualize the parenchymal enhancement of the prostate gland. To confirm the parenchymal enhancement of the prostate, C arm computed tomography (CT) angiography (Siemens, Erlangen, Germany) was performed from the prostatic arteries with hand injections of diluted contrast (1:4) when necessary.

The DSA images were reviewed by 2 interventional radiologists and discordant opinions were resolved by a third senior interventional radiologist. Patient age, prostatic artery (PA) origin and number of each pelvic side, procedure time (PT) and fluoroscopy time (FT) were recorded. Additionally, the PT was calculated for each pelvic side. The patients were divided into 3 groups according to their ages, as ≤68 years, 69–79 years, and ≥80 years. Prostatic arteries were classified using the de Assis/Carnavale classification (10) in 5 groups, comprising type 1: PA originating from the anterior division of the IIA, from a common trunk with the superior vesical artery (SVA); type 2: PA originating from the anterior division of the IIA, inferior to the SVA origin; type 3: PA originating from the obturator artery; type 4: PA originating from the internal pudendal artery (IPA); and type 5: less common origins of the PA, including from an accessory IPA, trifurcation or quadrification of the IIA anterior division, the inferior epigastric artery, the posterior division of the IIA, or from the distal segment of the IPA. The incidence of each anatomic type was recorded. In pelvic sides with more than one PA, the branch feeding the central gland was included. Pelvic sides with technical failure of IIA catheterization due to severe atherosclerosis or occlusion were excluded from the study. Moreover, prostatic arteries with occlusion or technical failure of catheterization due to severe atherosclerosis were excluded from the study, as confirmation of prostate gland enhancement could not be done in these pelvic sides.

### 2.1. Statistical analyses

The incidence of each anatomic type was calculated. The relationship of the PT and FT with patient age was compared using the Kruskal-Wallis test and ANOVA, respectively. The relationship of the PT and PA origin was analyzed using the Kruskal-Wallis test. IBM SPSS Statistics for Windows 22.0 (IBM Corp., Armonk, NY, USA) was used and P < 0.05 was accepted as statistically significant.

## 3. Results

**Figure 1:**
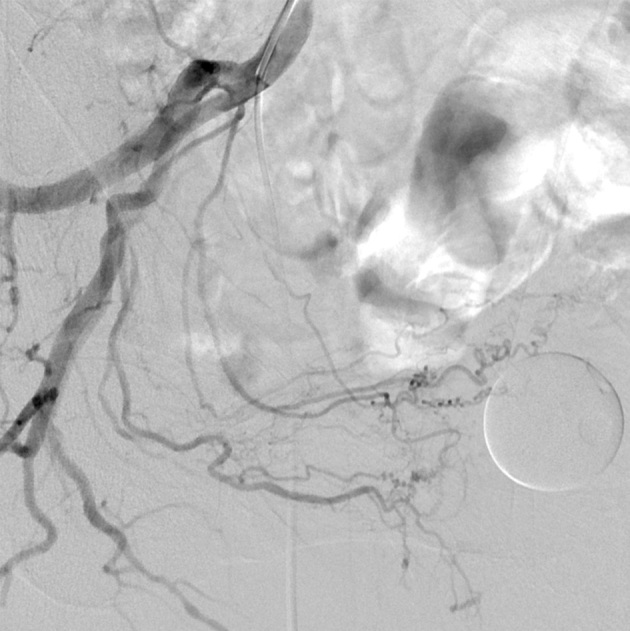
Ipsilateral oblique view of the right IIA angiography showing the type 1 anatomy of the PA arising from a common trunk with superior vesical artery.

**Figure 2:**
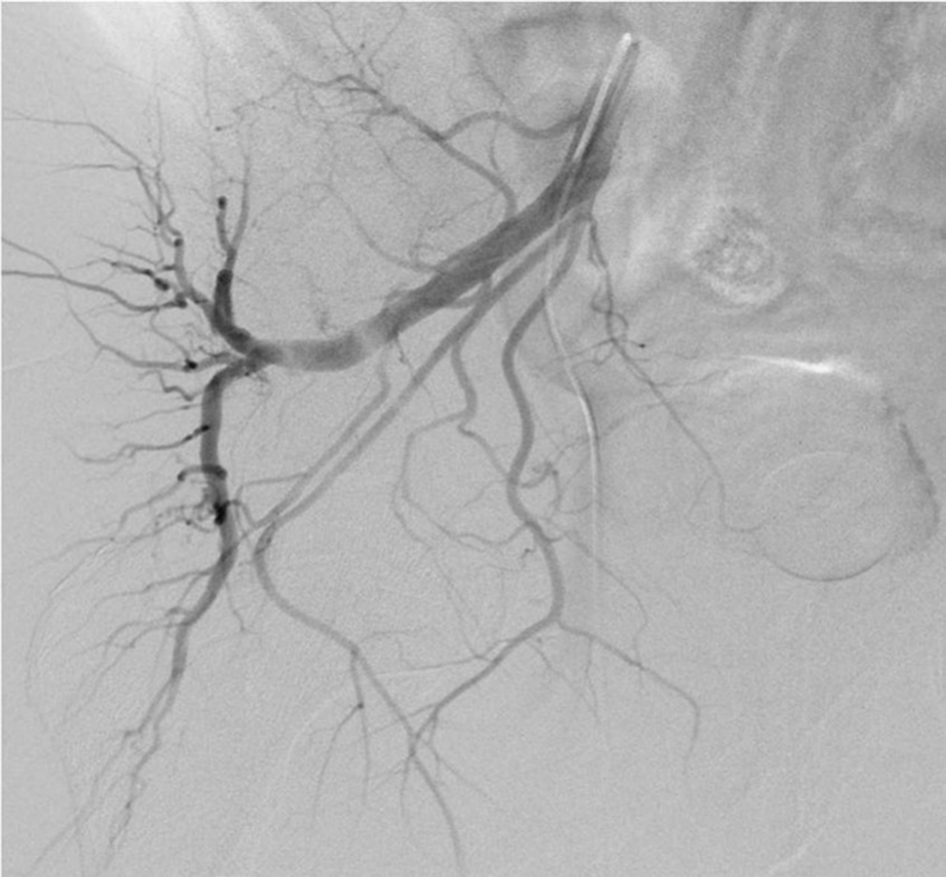
Ipsilateral oblique view of the right IIA angiography showing the type 4 anatomy of the PA arising from the internal pudental artery.

In the 68 PAE procedures, 4 patients (n = 8 pelvic sides) were excluded from the study due to technical failure of bilateral catheterization of the IIA/PA. In the remaining 64 procedures (n = 128 pelvic sides), 9 pelvic sides were excluded (n = 3 IIA occlusion, n = 6 PA occlusion) from classification. In the remaining pelvic sides (n = 119), double vascularization was seen in 3 pelvic sides (2.5%). A total of 119 PAs were classified into types 1–5. Among them, the most common origin was type 1 (n = 43, 36.1%) (Figure 1), followed by type 4 (n = 34, 28.6%) (Figure 2), type 3 (n = 22, 18.5%), and type 2 (n = 13, 10.9%). Type 5 anatomy was seen in 7 patients (5.9%) (Table 1). Among those of type 5, the most common origin was observed as PA arising from IIA anterior division quadrification (n = 4, 3.4%), followed by inferior gluteal artery (n = 3, 2.5%).

**Table 1:** Classification of the PA origins.

PAO	Type 1	43 (36.1%)
	Type 2	13 (10.9%)
	Type 3	22 (18.5%)
	Type 4	34 (28.6%)
	Type 5	7 (5.9%)

PAO: prostate artery origin.

The mean age of the patients was 72.1 years (SD: ±10.25). The median FT was 30 min (range: 8.9–84.8 min). The median PT was 57.3 min (range: 19.9–228.6 min). The median PT according to the types of PA were as follows: 1) type 1: 30.7 min, 2) type 2: 26.2 min, 3) type 3: 23.7 min, 4) type 4: 26.2 min, and 5) type 5: 38.3 min (P > 0.05). When each pelvic side was compared separately, the shortest PTs on the right and left pelvic sides were types 4 and 3, respectively. However, this was also not statistically significant (Table 2). When both the PT and FT were compared based on age, the FT had a tendency to increase as the age of the patient increased. However, this was not observed in the PT and neither were statistically significant (Table 3). 

**Table 2:** Comparison of the PA origins and procedure times.

	Origin of the PAs	Procedure time (min)		
		Median	IQR	*P-value
Left pelvic side	Type 1	36.3	33.1	0.555
	Type 2	47.1	45.5	
	Type 3	28.4	11	
	Type 4	35.3	36.3	
	Type 5	44.2	24.7	
Right pelvic side	Type 1	25.9	26.5	0.517
	Type 2	19.8	16.1	
	Type 3	19.4	18.2	
	Type 4	15	12.5	
	Type 5	29.7	33.7	
Total	Type 1	30.7	42	0.337
	Type 2	26.2	32.3	
	Type 3	23.7	16	
	Type 4	26.2	27.5	
	Type 5	38.3	33	

Results based on the *Kruskal-Wallis test. IQR: interquartile range.

**Table 3:** Fluoroscopy and procedure times between the age groups.

Age group	Fluoroscopy time (min)			Procedure time (min)		
	N (%)	Mean	± SD	N (%)	Median	IQR
≤68	18 (29.5)	30.7	16.2	19 (29.7)	51.2	31.5
69-79	27 (44.3)	35.1	15.7	29 (45.3)	61.3	47
≥80	16 (26.2)	39.8	20.3	16 (25)	61.2	49.1
Total	61 (100)*	35	17.2	64 (100)	57.3	42.2
P-value	0.315**			0.512***		

*The fluoroscopy times of 3 patients were missing. Results based on **ANOVA and the ***Kruskal-Wallis.

## 4. Discussion

From a clinical and imaging point of view, the de Assis classification is the simplest and most reproducible classification of this complex vascular system. Based on this classification, it was also possible to easily recognize the branching patterns of the IIA and PA, although the most common variation was PA arising from a common trunk with the SVA (type 1) in the current study cohort as opposed the study of de Assis et al. [10]. Type 4 (PA arising from the IPA) was the most common origin for PA in their study (31%), followed by type 1 (common trunk with SVA) origin (28%). Bilhim et al. also found similar results, as the most common origin of PA arising from IPA (type 4) in their study of 150 pelvic sides, followed by type 1 [11]. 

Wang et al. studied 318 PAs and found that the most common origin of the PAs was from a common gluteal-pudendal trunk with a SVA of 37% [12]. Although the most common origin of the current study cohort was similar with that of their study, their second most common origin was type 2 (31%) (from the anterior division of the IIA), whereas type 2 origin was seen only in 10% of the patient group herein. In cadaveric studies, the most common origin of PAs arose from the anterior division of the IIA, and was defined as the prostatic-vesical artery [5,13]. The difference in the incidence and order of the types of PA origins in the current study cohort may have been a result of the unique racial differences in the Turkish population. However, it should be kept in mind that the current study cohort consisted of a selection bias, as all of the data were obtained from patients with BPH, so the findings may not have reflected normal prostate glands. Further studies with a higher number cohorts are necessary to confirm these population-based variations.

In the current study cohort, double vascularization was only seen in 3 pelvic sides (3.2%), which was a lower incidence than the rates previously reported [7,10,11,14]. In a cadaveric study of 36 hemipelvises, double or triple PAs were reported, at an incidence of 22% [13]. The lower incidence of 2 independent PAs detected in the current study could be explained as the result of the different techniques that were used and/or as the result of racial differences. 

In the study cohort, no statistical significance were found between the PA origin types and the PT, which may have been due to the relatively small sample size. Nevertheless, in the current study cohort, when type 5 anatomy was excluded, the findings of type 3 anatomy, with a lower PT, and type 1 anatomy, with higher PT rates, still had clinical importance for interventional radiologists. Type 3 origins have a wider angle of origin and are easier to catheterize with microcatheters, whereas the angle of the origins in the type 1 anatomy are sharper than the other types, making them more difficult to catheterize, which is important, so that interventional radiologists performing the procedure will be prepared technically for possible challenging catheterizations. In the literature, studies investigating the factors affecting PAE procedure difficulty reported a statistically significant influence of the PA origin of type 3 anatomy with a lower FT, especially in comparison with type 1 anatomy, which also led to an increase in the dose area product [15,16], even though dose area products were not involved in the current study and the FT for each pelvic side was not available in the study cohort.

Type 5 anatomy was defined as origins other than types 1–4, and the most common variation in the current study cohort within the type 5 anatomy was origins from the IIA, such as quadrification. A frequent and important variation within the type 5 anatomy is PA arising from an accessory IPA [3], which was not observed in the current study cohort. This variation is of importance, as it may cause nontarget embolization to the pudendal territory.

Observationally, the FT times increased with age in the current study cohort, although this was not statistically significant. Age has a role in increasing iliac artery tortuosity and pelvic atherosclerosis [17], and although these covariates were not included in the current study, previous studies have reported increased FT and dose area products in the presence of tortuosity and atherosclerosis, even though age was not a significant predictor in the technical outcome [16].

Limitations of this study were the number of patients and its single-center retrospective nature. Moreover, an anatomic correlation with cadavers was not possible. Another limitation mentioned previously was that all of the data were obtained from patients with BPH; hence, the findings may not have reflected normal prostate glands. Further studies from other centers are necessary to confirm these population-based variations.

In conclusion, defining the origins and directions of PA is essential before PAE. Following a standard classification system and being aware of the most common types in each population will make the procedure faster and safer. 

## Informed consent

The study was approved by our institutional review board and informed consent was taken from all of the patients before any of the procedures were conducted (GO 19/312). 
